# The Homœopathic Medical Doctrine, or Organon of the Healing Art; a New System of Physic, from the German of Hahnemann

**Published:** 1833-10-01

**Authors:** 


THE HOMŒOPATHIC MEDICAL DOCTRINE, OR ORGANON OF THE HEALING ART; A NEW SYSTEM OF PHYSIC, translated from the German of *S. Hahnemann.* By *Charles H. Devrient*, Esq., with Notes, by *Samuel Stratten*, M.D. Octavo, pp. 332. Dublin and London, July, 1833.

HAVING reviewed the original Work in No. XXXIII., for July, 1832, we shall dedicate but a very short space to a notice of the translation.

A very cursory glance at this work has convinced us that it is founded in the grossest errors that were ever attempted to be palmed on a credulous public, in the garb of solemn truths. The fundamental principle, or rather *falsity*, of homœopathy is, "that certain medicines, when administered in a healthy state of the system, produce certain effects, and that the same medicines are to be used when symptoms similar to those which they give rise to occur in disease." Now let us look at a few of the *facts* on which this doctrine is based. The homœopathists say that they experimented upon people in *perfect health*, and by this artful set out they think that they cannot be contradicted, except by a train of experiments similarly conducted—which very few will take the trouble to make—and which still fewer have done hitherto. But this manœuvre may not, perhaps, serve their turn. We would first ask the homœopathists, however, where they found people, *in perfect health*, willing to undergo a train of experiments which actually rendered them ill? It was probably on their own persons that the experiments were made—and it is very easy *then* to suppose that they turned out exactly as the homœopathists desired and expected! Thus, a disciple of the great "ORGANON" took a grain of sulphate of quina three times a day; and what was the result? Why, in the course of a few days, the *appetite* was lost—nausea set in—then cold along the spine—rigor—reaction—perspiration!! In short, a regular ague-fit was induced by ten or fifteen grains of sulphate of quinine, taken in the course of four or five days!!! Was there ever such a ridiculous statement laid before a learned profession! It is to be remembered that this was not an occasional occurrence in one or two individuals (which might happen if so much flour were administered), but the inference is evidently implied, that the above is the regular and constant effect of quinine! So that, if a student wishes to see an intermittent—not a very usual sight in this country—he has only to produce one by a few grains of quinine—and then cure it by the same remedy—verifying the exploits of the fabled spear, which always healed the wound it had inflicted! The truth is, that the general effects of quinine on the constitution are the very reverse of what the homœopathists have stated them to be. Under such doses as above, the appetite is increased—a sense of heat is produced, not coldness—and perspiration, as well as all the other secretions, are diminished. In this, as in all other cases, the homœopathists have singled out a miserable *exception*, and paraded it as the *general* rule—without exception! Were there ever such error and folly seen united before? Here, then, the premises being false, the inferences fall to the ground as nullities.

*Again.* To prove that "*similia similibus curantur*," MERCURY is brought on the stage. Mercury cures syphilis; but the same metal "produces symptoms, *local* and constitutional, so closely resembling the poison of the lues venerea, that medical practitioners find it very difficult, nay, sometimes impossible, to distinguish one disease from another." Does the candid homœopathist admit that such effects of mercury are very rare, when we consider the number of people who take mercury—or that they occur only in particular constitutions, especially in those who have also the syphilitic poison lurking in their veins? Oh! no. The above effects are adduced as the general, the universal effects of mercury—*without apparent exception.* Among the ten thousand people to whom Mr. Abernethy administered the blue-pill, how many, may we suppose, had regular chancres (the local symptoms) produced by the mercury? We dare to say that there was not one! But to go from suppositions to realities. What analogy has the operation of mercury, and that of syphilis, on the human frame? None whatever. Mercury acts on the whole glandular system, increasing the secretions and excretions, producing, if carried to a certain extent, salivation. Does the syphilitic poison produce these effects? No. Besides antimony, sarsaparilla, and many other medicines, of opposite qualities, will cure syphilis. Nay, starvation and keeping in bed will do the same. Here, again, the homœopathic premises being *false,* the deductions are equally so.

*Thirdly,*

"Nitric acid is generally recommend in cutaneous diseases; the internal use of this remedy in a very dilute form, produces scaly eruptions over the surface of the body; and the external application of a solution, in the proportion of one part acid to one hundred and twenty-eight parts of water, will produce inflammation and ulceration of the skin. These observations would lead to the conclusion, that nitric acid cures cutaneous diseases by the faculty it possesses, of producing a similar disease of the skin."—*Preface,* v.

We verily believe the assertion that dilute portions of nitric acid, taken internally, produces scaly eruptions, is a mere assumption—or, if it ever happen, that it is, like the preceding facts, exceptions to general rules. In the East Indies, where diluted nitric acid used to be exhibited, on a large scale, for syphilitic affections, and as a tonic after bowel and liver complaints, we do not remember to have seen a single instance of the kind, so confidently trumpetted forth as a general rule by the advocates of homœopathy. As to the external use of the acid, what would the ghost of Scott say, if he were to rise from his watery grave in the pacific ocean? He who employed more of this acid to the feet and legs of his patients, than would float a three decker in one of the East India docks, has told us nothing of this homœopathic piece of intelligence!

*Fourthly.* Nitrate of potash, say the disciples of Hahnemann, *taken in small doses,* produces "frequent desire to make water, accompanied with *pain and heat.*" This is not a *fact,* but a fiction. It is a mere gratuitous assertion to preface, homœopathically, what is truth—viz. "when this state of the urinary system exists as a consequence of disease, or the application of a blister, a very dilute solution of the same remedy has been found beneficial." Here, again, the premises are *false,* and the inferences, though true, as to fact, are inconsequential as to doctrine.

*Fifthly.* We are told by the promulgators of the ORGANON, that hyoscyamus *ordinarily* produces "vertigo, delirium, stupefaction, and somnolency."

Here is one of the candid statements of the homœopathists! Hyosciamus, in poisonous doses, or in peculiar idiosyncrasies of constitution, may produce the foregoing effects—in the same way that turtle-soup may produce apoplexy, or wine insensibility. All medicines are poisons, when administered in inordinate doses, and the most wholesome food may destroy life, when taken to excess.

" The internal use of hyosciamus is followed by mental aberration, the leading features of which are jealousy, and irascibility. When these hallucinations exist, this remedy is indicated." *Pref.* vi.

Thus hyosciamus first produce jealousy and mental aberration—and then removes the same symptoms! This is blowing hot and cold with the same breath truly.

" Opium in general causes drowsiness, torpor, and deep sleep, and yet this remedy in small doses removes these symptoms when they occur in disease." *Pref.* vi.

What practitioner would think of exhibiting opium, in any doses, for " drowsiness, torpor, and deep sleep?" Here is another gratuitous assertion, not only without proof, but against all experience.

" Sulphur is a specific against itch; notwithstanding which, when it is administered to healthy individuals it frequently excites a pustular eruption resembling itch in every particular." vi.

" A mixture (says the translator) composed of one drop of hydrocyanic acid and eight ounces of water, administered in a drachm dose, has produced vertigo and anxious breathing. Vomiting has followed the use of the sixteenth of a grain of emetic tartar; narcotism the twentieth of a grain of muriate of morphia; and spirit of ammonia, in doses of one drop, acts on the system as a stimulant." *Pref.* vii.

Admitting the truth of the above statements, what then? Why, where one person experienced such effects, 999 would experience no effect whatever, from such doses of prussic acid, antimony, opium, or ammonia!

It is upon such facts as these that the new doctrine rests! It is difficult to imagine how a physician, in the 19th century, could gravely enunciate such a series of absurdities, errors, and downright falsities, as the basis of a doctrine that was to overturn all former observation, experiment, and experience! But it is still more wonderful that other physicians, in his own and in other countries, should have been led to adopt such preposterous notions. That homœopathy will ever take root in this country, we may safely venture to deny—except as a branch of trade to make money among our wise aristocracy, the patrons of St. John Long and his tribe! That money will be extracted out of the pockets of our gentry, by these fooleries, we have no doubt. It is not more difficult than the extraction of quicksilver from the heads of his patients, by the Harley Street Quack. Lords will be found to verify the cures of homœopothy, as easily as to certify the quicksilver extraction of Long. At the very time when we were drawing up this notice of the Organon, we were summoned to a patient who had been under the hands of one of the disciples of homœopathy, during two or three months, for the cure of an organic affection. The patient was furnished with a vial not near so wide as a common silver pencil case, and about one-fourth its length, containing more than fifty pills, scarcely the size of grains of sand.

One of these was to be placed on the tip of the tongue, twice a day, and there allowed to dissolve. The complaint continued unabated—and so did the doctor's visits every second or third day—without any charge however for the medicine, but only a common fee for his visit! It so happened, however, that the patient was seized with a severe bowel complaint; and cholera being apprehended, it was thought prudent to call us in, without saying a word about the homœopathist, who still continued his attendance, equally ignorant that another physician was employed. It was necessary to use very active measures, and even to make the mouth sore with calomel and opium. Afterwards, the secret came out. The homœopathist, when he found a new disease had supervened, thought it necessary to leave a new remedy, every day—a powder that required a magnifying glass to be distinguishable at all. The powders remained untouched, and when the patient was getting well, under the influence of mercury and opium, the doctor rubbed his hands, and, with a very knowing look, told the patient that, had she been under the care of some of the London physicians, all this time, her diarrhœa would certainly have turned to cholera, and death would have been the consequence. This wonderful homœopathic cure will, if successful, (for that is not certain,) make a figure in the next edition of the ORGANON.

In respect to the ORGANON itself, we have waded through the 292 sections of which it is composed; and such a tiresome tissue or jumble of unintellible gibberish, erroneous *reasoning* (God save the mark!), and transcendental balderdash, it was never before our hard fate to encounter! This is saying a good deal, considering that we have served an apprenticeship of some sixteen years to the noble art and mystery of viewing and reviewing, sifting and searching, weighing and winnowing, the productions of the medical press! Of the ORGANON, it may be truly said that there is "nil simile aut secundum," in medical literature. But, being a HOCUS POCUS of mysterious nonsense, we have little doubt that, for a season, it will prove a profitable engine in the hands of those who choose to work it to their own advantage. It is an admirable system for high-lifed humbug. The medicines are all secret—all to be furnished, prepared, and exhibited by the homœopathic doctor. The doses, too, are infinitely smaller than grains of mustard-seed, and have no other taste than that of sugar. All incurable diseases are to be removed or annihilated with certainty—"cito, tutè, ac jucunde." Need we say more? St. John Long and Dr. Morison may shut up shop. Their burning liniments and cholera-facient pills are all on the "*contraria contrariis*" SYSTEM, and, consequently, out of date. The Apothecaries' Hall—the great chemists—nay, every little chemist and druggist, must soon go on the parish, since the five-hundredth part of a grain of quinine will be a large dose—and a pint of BATTLEY will serve all London for twelve months! But to be serious:—If doctors can be such fools as to attempt the cure of *real* diseases by homœopathic remedies—and if patients can be such fools as to trust their bodies in such hands, why then they are fools on both sides. There will be plenty of "*similia similibus*"—but a woeful lack of the "*curantur.*"

